# Immediate access arteriovenous grafts versus tunnelled central venous catheters: study protocol for a randomised controlled trial

**DOI:** 10.1186/s13063-015-0556-x

**Published:** 2015-02-08

**Authors:** Emma Aitken, Colin Geddes, Pete Thomson, Ram Kasthuri, Mohan Chandramohan, Colin Berry, David Kingsmore

**Affiliations:** Department of Renal Surgery, Western Infirmary, Dumbarton Road, Glasgow, G11 6NT UK; School of Medical, Veterinary and Life Science, University of Glasgow, University Avenue, Glasgow, G12 8TA UK; Department of Nephrology, Western Infirmary, Dumbarton Road, Glasgow, G11 6NT UK; Department of Radiology, Western Infirmary, Dumbarton Road, Glasgow, G11 6NT UK; British Heart Foundation Cardiovascular Research Centre, Institute of Cardiovascular and Medical Sciences, University of Glasgow, University Avenue, Glasgow, G12 8TA UK

**Keywords:** Tunnelled central venous catheter, Arteriovenous graft, Vascular access, End-stage renal disease

## Abstract

**Background:**

Autologous arteriovenous fistulae (AVF) are the optimal form of vascular access for haemodialysis. AVFs typically require 6 to 8 weeks to “mature” from the time of surgery before they can be cannulated. Patients with end-stage renal disease needing urgent vascular access therefore traditionally require insertion of a tunnelled central venous catheter (TCVC). TCVCs are associated with high infection rates and central venous stenosis.

Early cannulation synthetic arteriovenous grafts (ecAVG) provide a novel alternative to TCVCs, permitting rapid access to the bloodstream and immediate needling for haemodialysis. Published rates of infection in small series are low.

The aim of this study is to compare whether TCVC ± AVF or ecAVG ± AVF provide a better strategy for managing patients requiring immediate vascular access for haemodialysis.

**Methods/design:**

This is a prospective randomised controlled trial comparing the strategy of TCVC ± AVF to ecAVG ± AVF. Patients requiring urgent vascular access will receive a study information sheet and written consent will be obtained. Patients will be randomised to receive either: (i) TCVC (and native AVF if this is anatomically possible) or (ii) ecAVG (± AVF).

118 patients will be recruited. The primary outcome is systemic bacteraemia at 6 months. Secondary outcomes include culture-proven bacteraemia rates at 1 year and 2 years; primary and secondary patency rates at 3, 6, 12 and 24 months; stenoses; re-intervention rates; re-admission rate; mortality and quality of life. Additionally, treatment delays, impact on service provision and cost-effectiveness will be evaluated.

**Discussion:**

This is the first randomised controlled trial comparing TCVC to ecAVG for patients requiring urgent vascular access for haemodialysis. The complications of TCVC are considered an unfortunate necessity in patients requiring urgent haemodialysis who do not have autologous vascular access. If this study demonstrates that ecAVGs provide a safe and practical alternative to TCVC, this could instigate a paradigm shift in nephrology thinking and access planning.

**Trial registration:**

This study has been approved by the West of Scotland Research Ethics Committee 4 (reference no. 13/WS/0087, 28 August 2013) and is registered with the International Standard Randomised Controlled Trial Number Register (reference no. ISRCTN80588541, 27 May 2014).

## Background

Vascular access dysfunction is the leading cause of morbidity amongst patients with end-stage renal disease (ESRD) and is a key performance indicator for haemodialysis service provision [1]. Complications of vascular access are responsible for over 20% of all hospitalisations in patients on haemodialysis and account for one-third of all inpatient renal bed use [2].

Autologous arteriovenous fistulae (AVFs) are the vascular access modality of choice [1,3] with fewer infective and thrombotic complications than the alternatives [1,4]: tunnelled central venous catheters (TCVCs) and prosthetic arteriovenous grafts (AVGs). The major limitations to native AVF usage are a 6 to 8 week “maturation” lag from creation to first cannulation and 30 to 50% early failure rate [5,6]. Unfortunately, due to a combination of late referral, primary access failure and acute presentation of renal failure, 40 to 50% of incident patients do not have a functioning AVF when they commence dialysis [3,7,8]. Given the unpredictable progression of decline in renal function to ESRD [9,10] and the fact that 30 to 35% of patients needing to start haemodialysis are “crashlanders” (known to the renal services for <90 days) [3], whilst desirable, it is not always practicably possible to achieve autologous vascular access for all patients prior to commencing haemodialysis. Current practice necessitates TCVC for these patients until definitive vascular access can be secured. Patients who began haemodialysis via a TCVC are more likely to remain with TCVC [11]; therefore, optimizing incident vascular access is vitally important.

TCVCs are associated with a significantly higher risk of infection, mortality, mechanical complications and other chronic problems such as immunosuppression, malnutrition and central venous stenosis than other forms of vascular access [1,12]. A recent Scottish study of 2,666 patients revealed a two- to threefold increased risk in mortality (all-cause mortality, cardiovascular or infection-related mortality) and a sevenfold increase in death from septicaemia with the use of TCVCs [12]. For these reasons, TCVC are considered an option of last resort.

AVGs provide an intermediate option between AVF and TCVCs, permitting earlier cannulation than AVF but lower bacteraemia rates than TCVC (0.6 per 1,000 dialysis days) [13]. The longevity and patency of AVGs are variable and almost certainly poorer than native AVF with 1-year primary patency rates ranging from 40 to 60% [14]. However, with aggressive re-intervention for the management of thrombosis, secondary patency rates of as high as 90% at 1 year can be achieved [15].

Traditional AVGs still require a delay of approximately 2 weeks from implantation to initial cannulation, compelling TCVC insertion as a stop-gap measure in the majority of cases. However, early cannulation arteriovenous grafts (ecAVGs), which permit immediate needling, may provide a novel alternative permitting TCVC avoidance in many cases [16]. The GORE**®** Acuseal™ (W.L.Gore Associates Ltd., Flagstaff, Arizona) vascular access graft is one such ecAVG. It is a tri-layer graft with unique sealing properties to permit safe cannulation within 24 hours of insertion. The tri-layer construction comprises an inner layer of heparinised expanded polytetrafluoroethylene, an outer layer of standard expanded polytetrafluoroethylene graft, and a central elastomeric layer [17]. This central layer gives the graft its unique “low bleed” properties and permits early cannulation, reducing the time to achieve haemostasis significantly compared to standard expanded polytetrafluoroethylene.

These unique properties of Acuseal™ and other ecAVGs make them an attractive alternative to TCVCs in patients requiring urgent vascular access for haemodialysis with theoretical benefits of reduced infection and avoidance of long-term TCVC complications such as central venous stenosis. In small series, the Acuseal™ graft has been shown to be beneficial in patients requiring urgent access for haemodialysis as a “bridge” to AVF maturation or transplantation, as well as in patients with pre-existing occlusion of their central veins [18]. A recent observational study conducted in our unit has indicated that ecAVG provide a practical, feasible and acceptable alternative to TCVCs in patients requiring urgent vascular access for haemodialysis [19]. Furthermore, cost-analysis has shown that the additional initial outlay costs of consumables can be offset against reduced septic complications, hospital admissions and bed days, with potential cost savings of nearly £1,000 at 6 months with ecAVG compared to TCVCs [20].

We now intend to test this hypothesis in a randomised controlled trial comparing TCVCs (± AVF) to ecAVG (±AVF).

## Methods/design

### Overview

This is a single centre randomised controlled trial. This study has been approved by the West of Scotland Research Ethics Committee 4 (reference no. 13/WS/0087) and is registered with the International Standard Randomised Controlled Trial Number Register (ISRCTN80588541). This study will be performed in keeping with the requirements of the Declaration of Helsinki.

### Hypothesis

The use of ecAVGs ± AVFs will reduce the rate of culture-proven bacteraemia at 6 months compared to the use of TCVCs ± AVFs in patients with ESRD requiring urgent vascular access for haemodialysis.

### Objectives and outcome measures

This study aims for a strategy of comparing ecAVG ± autologous AVF (if anatomically suitable) to TCVC ± autologous AVF in patients requiring urgent vascular access for haemodialysis. The primary outcome measure is culture-proven bacteraemia at 6 months. Secondary outcomes include culture-proven bacteraemia at 12 and 24 months; primary, secondary and functional patency rates at 3, 6, 12 and 24 months; stenosis, thrombosis and re-intervention rates (including the need for urokinase infusions, AVG declotting procedures and TCVC replacement); other complications, including local infection, suspected access-related infection, steal syndrome, cardiovascular compromise, central venous stenosis; mortality at 6, 12 and 24 months, quality of life (assessed by EQ-5D) at 6 months; future sites of vascular access; re-admission rates and bed day utilisation and efficiency and efficacy of dialysis (urea reduction ratio, access flows, and recirculation). Additionally delays to treatment and the impact on service provision (access to theatre, and so forth) will be assessed to evaluate the practicalities of each treatment regimen. Cost-utility analysis will also be performed. Definitions of patency and other access-related complications will be as described by Sidawy and colleagues [21].

### Study centre

The study will be conducted at the Western Infirmary, Glasgow, UK. This university teaching hospital provides a tertiary referral vascular access service for 650 haemodialysis patients in the West of Scotland. We perform approximately 400 vascular access procedures, including 100 complex access procedures, annually. We have a wealth of experience using ecAVGs with published local outcomes highlighting our current practice [18-20]. The necessary volume of clinical cases, presence of clinical expertise and equipment required for this study is well established in this unit.

### Patients and enrolment

All patients will be admitted under the care of the Nephrology Team at the Western Infirmary. Patients requiring urgent vascular access for haemodialysis will be assessed by their clinical team. Temporary venous catheter will be inserted to permit life-saving haemodialysis if required. The clinical team will then refer the patient to the Vascular Access Co-ordinator for TCVC insertion and definitive vascular access creation.

All patients referred for TCVC insertion will be assessed by a member of the research team within 6 hours of referral. All adult patients (≥18 years old) with ESRD requiring urgent vascular access for haemodialysis will be eligible to participate. Patients will be excluded if they have significant cardiorespiratory co-morbidities or peripheral vascular disease precluding anaesthesia or ecAVG creation, a brachial artery <2 mm diameter on duplex ultrasound, significant and untreated systemic sepsis with positive blood cultures, women who are pregnant or breast-feeding, lack of capacity or ability to provide informed consent or if they decline to participate.

### Consent

The process of consent will be in accordance with the Declaration of Helsinki. All suitable patients will be fully informed that they are being asked to participate in a research study. The procedures involved in the study, and the chances of being assigned randomly to one of two groups will be explained in person and via an information sheet approved by the West of Scotland Ethics Committee. A signed consent form will be obtained from each patient and retained by the investigators. Patients will be made aware of their right to withdraw from the study at any time without adverse effects on their clinical care.

### Pre-operative management

All patients will undergo Duplex ultrasound of both arms. Both the venous and arterial tree will be assessed and a pre-operative plan made to site both ecAVG and native AVF. Wherever possible, care will be taken to preserve all sites for future autologous access and the site of native AVF will always be favoured in the non-dominant arm and distally first. ecAVG will be placed to accommodate optimal AVF placement. For example, a native left radiocephalic fistula and right forearm loop or right brachioaxillary graft would be favoured in a left-handed patient with good native vessels and no previous vascular access; whilst revision of an existing occluded left brachiocephalic fistula with outflow stenosis using an interposition ecAVG and contralateral elbow AVF would be considered in an elderly patient with poor vessels and occluded existing AVF.

### Randomisation

A computer generated 1:1 allocation sequence will be created by an independent operator who is not directly involved with the study. Allocation concealment will be achieved using sequentially numbered sealed opaque envelopes.

### Blinding

Due to the nature of the treatment and any subsequent interventions, it is not possible to blind either patient, surgeon or investigator to the treatment allocation.

### Treatment strategies

Patients will be randomized to receive either ecAVG ± AVF or TCVC ± AVF.

#### TCVC ± AVF

TCVC ± AVF reflects standard practice at our institution. Patients randomized to receive TCVC ± AVF will be referred to the Interventional Radiology Department for TCVC insertion (either by a radiologist or nurse specializing in TCVC insertion).

Tunnelled Ash Split**®** (Medcomp, Harleysville, PA, USA) 14Fr double-lumen polyurethane haemodialysis catheters will be inserted with 280- or 320-mm catheters used for right- and left-sided cannulation, respectively. Catheters will be inserted via a Seldinger technique under image guidance. A standard catheter care protocol will be employed throughout the study period. This demands complete sterile precautions during catheter insertion and upon manipulation of the hub. Following catheter hub manipulation, the skin surrounding the insertion site is soaked with chlorhexidine solution prior to a sterile dressing being applied. An interdialytic lock with heparin 5,000 iU/ml to the internal volume of the catheter will be employed.

First haemodialysis via the TCVC will be performed by trained nursing staff within the InPatient Renal Unit at the Western Infirmary. A record of any difficulties or complications with initial dialysis will be made. The timing of the first dialysis via the TCVC will be made at the discretion of the nephrology team, as will the need for anticoagulation. A record of these factors will be kept.

The patient will be discharged to dialyse at their Outpatient Dialysis Unit at the discretion of the nephrology team. Any problems that subsequently develop with the TCVC will be referred to and managed by the nephrology team at the Western Infirmary as is standard practice. These will be recorded in the patient’s electronic patient record and reported to the Principal Investigator.

#### ecAVG ± AVF

Patients randomised to the ecAVG ± AVF arm of the study will be immediately assessed by a member of the anaesthetic team. An operative slot will be found within either emergency or elective theatres at the Western Infirmary for ecAVG creation within 24 hours.

All patients will be treated with pre-operative prophylactic vancomycin 1 g intravenously (or teicoplanin if the patient is vancomycin allergic). ecAVG insertion will be performed by a single operating surgeon either under supraclavicular block or general anaesthetic.

The patient’s skin will be prepared with alcoholic betadine, draped in a standard fashion and an Ioban™ skin covering (3 M Healthcare, Bracknell, UK) applied to maintain strict asepsis. The vessels will be exposed and controlled in a standard fashion. The Acuseal™ graft will then be tunnelled in the subcutaneous fat using standard tunnelling tools. A 4-cm longitudinal venotomy will be performed and the graft spatulated at the venous end in an attempt to minimize venous stenosis. The arteriotomy will be a standard size to accommodate the graft. Arterial and venous anastomoses will be performed using continuous 5.0 Prolene. Collatamp™ (Tribute Pharmaceuticals, Milton, Ontario, Canada) will be inserted into the wounds prior to closure to minimize the risk of infection. Drains will be placed at the surgeon’s discretion.

Post-operatively the patient will be managed within the Inpatient Renal Unit of the Western Infirmary. First cannulation of the ecAVG will be performed by our trained dialysis nursing staff in agreement between the Nephrology and Surgical Teams as the patient’s clinical condition dictates. This may be as soon as 30 minutes post-operatively.

Sharp needles (17 G), low flows (200 to 250 ml/min) and minimal heparin will be used for first cannulation of ecAVGs. Full aseptic technique will be used for cannulation and direct pressure applied at the needle sites for at least 10 minutes after the needles have been removed. These same techniques will be used for the first 2 weeks of cannulation. Thereafter, higher flow rates may be used if necessary to achieve adequate dialysis clearance. Success and complications of dialysis sessions and cannulation will be recorded. At least two successful cannulations of the ecAVG will be performed prior to discharge to the Outpatient Dialysis Unit. All patients will continue on intravenous vancomycin for a week post-operatively. Heparin, warfarin and anti-platelet agents will be administered at the discretion of the operating surgeon. All patients who re-present with thrombotic complications will be anti-coagulated with warfarin (international normalised ratio 2–3) unless contraindicated.

Should the patient develop any problems with their ecAVG upon discharge they will be reviewed immediately by a member of the Renal Surgical Team at the Western Infirmary as is standard practice. In the event of thrombosis of ecAVG, aggressive attempts will be made by both the surgical and radiological teams to salvage the dialysis access as is standard practice. All complications will be recorded within the patient’s electronic case record and reported to the research team.

#### Autologous AVF

Patients in both treatment arms will also undergo creation of an autologous AVF (if this is anatomically possible) at the first available opportunity. The ecAVG/TCVC will continue to be utilized for haemodialysis until the AVF is mature enough to cannulate. The decision to undertake first cannulation of the AVF will be taken by the clinical team (normally ~6 weeks after creation) and recorded within the patient’s electronic patient record.

Once the patient is successfully dialyzing via their AVF, the fate of an ecAVG, which is no longer required, will be decided after discussion between the patient and surgical team. In the majority of cases it will be left *in situ* but can be removed or ligated if required/wished. TCVCs will be removed by the surgical team after six successful AVF cannulations as is standard practice.

### Follow-up and data collection

All patients will be reviewed by the research team on day 1, day 7 and 3, 6, 12 and 24 months following insertion of ecAVG/TCVC and 6 weeks following creation of AVF. Additionally surveillance of ecAVG will be performed by both ultrasound and venography at 3-monthly intervals. Data will be collected prospectively from the operative notes and Scottish Electronic Renal Patient Record.

Patient demographics will be obtained including age, gender, ethnicity, weight, body mass index, dialysis status, current access modality and co-morbidities. Details of operative surgery, anaesthetic, site of ecAVG, TCVC and AVF and perioperative complications will be recorded. Date and details of first cannulation/utilisation of ecAVG/TCVC will be recorded in the case report form along with the date and nature of any complications. Quality of life will be assessed at 6, 12 and 24 months. All data will be anonymised. Case report files will be archived in a locked facility for a period of 5 years.

### Criteria for discontinuation

Every effort will be made to retain patients in the trial and to minimise withdrawals. However, any severe or life-threatening event will be sufficient to remove a patient from the study. Data collected prior to the point of withdrawal from the study will be retained. Patients may request to be withdrawn from this study at any time. Reasons for withdrawal will be documented. Intention-to-treat analysis will be performed.

### Sample size and statistical considerations

Locally, 24% of TCVCs result in systemic bacteraemia within 6 months [19]. In order to calculate sample size, we made the following assumptions: type 1 error (α) was set at 0.05 and type 2 error (β) at 0.8. Therefore, if we assume a systemic bacteraemia rate in the TCVC arm of 24% at 6 months and propose a bacteraemia rate of 5% at 6 months in the ecAVG arm, then 53 patients are required in each group. In order to account for attrition of around 10%, we aim to recruit 118 patients. This magnitude of difference between the two treatment arms is considered to be clinically significant and realistic given the published rates of systemic bacteraemia for ecAVG in our institution [18].

The null hypothesis for this study is that there is no difference between systemic bacteraemia rates at 6 months in patients who require urgent vascular access for haemodialysis treated with TCVC ± AVF and those treated with ecAVG ± AVF.

Descriptive statistics will be used to describe continuous variables. Results for continuous variables will be reported as mean (± standard deviation) or median (interquartile range). Assuming normal distribution, treatment groups will be compared using a student’s t-test or, if data are found to not be normally distributed, a Mann–Whitney U-test will be used. Analysis will be performed on an intention-to-treat basis. Additionally, a cost-consequence analysis will be conducted, comparing the costs (for example, treatment, procedures, hospital stay, and so forth) and the consequences (for example, health outcomes such as bacteraemia episodes, mortality, and so forth) of both treatment arms.

Based on the results of our recent observational study [19] in which 53 eligible patients presented in a 6-month period, it is anticipated that recruitment will take approximately 18 months. Data collection will continue for 2 years beyond the date of last recruitment, though the primary outcome will be assessed after 6 months.

### Adverse event reporting and safety

All adverse events will be fully recorded in the medical records and on the study case report forms. A Data Monitoring Committee comprising of the Chief Investigator, trial statistician and an independent nephrologist will convene at the mid-point of the trial to evaluate recruitment and data collection. The Data Monitoring Committee will monitor adverse events and make recommendations as required. An interim analysis of results will be performed at the mid-point of the trial.

All adverse events will be evaluated by the Chief Investigator for severity, expectedness and causality. Any serious adverse events (SAEs) will be reported to the main Research Ethics Committee and sponsor where, in the opinion of the Chief Investigator, the event was related (resulted from administration of any of the research procedures) and unexpected (not listed in the protocol as an expected outcome). SAEs will be reported using the National Research Ethics Service SAE report form for Clinical Trials of Non-Investigational Medical Products.

Both TCVCs and AVGs are used routinely in clinical practice with minimal complications. The most common risk of TCVC usage is systemic bacteraemia, quoted in the range of 1.4 to 1.8 per 1,000 catheter days [4,12,13,22]. Most commonly, Gram-positive organisms, for example *Staphylococcus Aureus*, cause catheter-related bacteremia [23]. Otherwise, catheter replacement for dysfunction (thrombosis and malposition) occurs in approximately 0.2 per 1,000 catheter days [23]. The well-described complications of pneumothorax and carotid artery puncture are very rare with image-guided TCVC insertion.

Similarly, operative complications of ecAVG, including steal syndrome and bleeding, are rare [18]. Infection rates are lower than TCVC (approximately 0.6 per 1,000 dialysis days in most published series). The most common infective organisms are Gram-negatives, which are more common in lower rather than upper limb accesses [24]. Thrombosis of ecAVG is the most common complication, with approximately half of ecAVGs requiring re-intervention during the first year [14]. With aggressive management strategies for thrombosis, however, it is possible to achieve secondary patency rates approaching 90% at 1 year [15].

Any adverse events relating to either procedure will be recorded by the staff performing the study, and necessary investigations, treatment or follow-up arranged thereafter.

### Limitations and potential solutions

Traditionally, recruitment in trials relating to vascular access can be difficult. In one recent large study, over 2,000 patients were screened to recruit just 225 [25]. This study had very strict inclusion criteria, which can be difficult to achieve in a heterogeneous patient population such as ESRD. It is not anticipated that such problems will be encountered in our study for several reasons: firstly, this will be an inclusive study (rather than having strict inclusion/exclusion criteria); secondly, a local pilot study [19] has demonstrated that 79 patients had TCVCs inserted over a 6-month period and therefore might be approached for inclusion in this study.

The power calculation for this study is based on a bacteraemia rate of 24% in the TCVC arm. This rate is higher than that described in many series, but is derived directly from our local pilot study [19]. It may be that these higher rates of infection relate to early post-insertion infection rates, rather than overall bacteraemia rates for static prevalent TCVCs.

## Discussion

The purpose of this trial is to investigate the hypothesis that, in patients requiring urgent vascular access for haemodialysis, the 6-month culture-proven bacteraemia rate is improved by a strategy of ecAVG ± AVF compared to TCVC ± AVF as this has not yet been demonstrated in a large randomised controlled trial.

Complications of vascular access are a leading cause of morbidity and mortality in patients with ESRD. The healthcare costs from a catheter-related bacteraemia range from $6,000 to $29,000 [26]. If the proposed strategy of ecAVG ± AVF is found to reduce culture-proven bacteraemia rates, it could result in significant reduction in healthcare costs and reduced morbidity and hospitalisation for patients with ESRD. This could lead to a paradigm shift away from TCVC in patients requiring urgent vascular access. Conversely if TCVC ± AVF is found to be superior, this finding would support our current strategy of TCVC usage in this patient cohort. Therefore, either a positive or negative result will help inform future practice regarding the optimal strategy of vascular access in patients requiring urgent haemodialysis.

## Trial status

Recruiting.

### Finance and indemnity

A small Investigator Led research grant from W.L. Gore Associates will assist in analysis of secondary end-point data. The funders have no role in the study design, data collection or analysis, decision to publish or preparation of the manuscript. NHS employed researchers will be covered for negligent harm through the NHS CNORIS indemnity scheme.

## Abbreviations

AVF: arteriovenous fistula
AVG: arteriovenous graft
ecAVG: early cannulation arteriovenous graft
ESRD: end-stage renal disease
SAE: serious adverse event
TCVC: tunnelled central venous catheter
